# Changes in Physical Activity Behaviour and Health Risk Factors Following a Randomised Controlled Pilot Workplace Exercise Intervention

**DOI:** 10.3934/publichealth.2017.2.189

**Published:** 2017-05-10

**Authors:** Naomi Burn, Lynda Heather Norton, Claire Drummond, Kevin Ian Norton

**Affiliations:** 1 Health and Social Care Institute, Teesside University, Middlesbrough Tees Valley, TS1 3BX, UK; 2 School of Health and Exercise Science, Faculty of Medicine, Nursing and Health Sciences, Flinders University of South Australia, Adelaide, South Australia, 5001; 3 School of Health Sciences, University of South Australia, Adelaide, South Australia, 5001

**Keywords:** workplace interventions, physical activity, health outcomes

## Abstract

**Background:**

Declining physical activity (PA) and associated health risk factors are well established. Workplace strategies to increase PA may be beneficial to ameliorate extensive sedentary behavior. This study assessed the effectiveness of two PA interventions in workplace settings.

**Methods:**

Interventions were conducted over 40 days targeting insufficiently active (<150 min/wk PA) and/or obese (BMI ≥ 30 kg/m^2^) adults; participants were randomly allocated to instructor-led exercise sessions either after-work (n = 25) or in-work (n = 23) with a 60 minPA/day common goal, or a wait-listed control group (n = 23). The programme commenced with low-moderate physical activities and progressed to high intensity game style activities by week six. Adherence and compliance were determined using both objective measures of daily PA time from HR monitors and self-report responses to PA questionnaires. Cardiovascular and metabolic risk factors were measured pre- and post-intervention. Changes across the study were analysed using Chi square and repeat-measures ANOVA.

**Results:**

Adherence rates (completed pre and post-testing) were not different between groups (76.0 *vs* 65.2%). Compliance for the instructor-led sessions was higher for the after-work group (70.4% *vs* 26.4%, respectively). Increased total PA and aerobic fitness, and decreased weight in both intervention groups were found relative to controls. The after-work group undertook more vigorous PA, and had greater weight loss and fasting blood glucose improvement, relative to in-work participants and controls.

**Conclusions:**

These workplace interventions resulted in rapid and dramatic increases in PA behaviour and important health benefits. Short, in-work PA sessions were less efficacious than longer after-work sessions.

## 1. Introduction

Physical inactivity is the fourth leading cause of death worldwide, ahead of obesity [1]. Workplaces are increasingly facilitating extensive periods of sedentary behaviour [2] with studies reporting that over 50% of daytime sitting occurs in the workplace [3]. Current Australian PA guidelines recommend 150–300 minutes of moderate-intensity PA per week for health benefits [4], and up to 420 min/wk is recommended for weight loss [5]. Growing evidence suggests workplace PA interventions can be effective in increasing PA behaviours and reducing weight among employees [6],[7]. Important elements of successful PA interventions, at least for the short-term, include convenience, exercise guidance, social interaction, on-going monitoring and feedback, counselling and/or education sessions, and programme variety [8],[9]. Notwithstanding, there are limited studies comparing different workplace-based environmental approaches to effect PA and health risk factor changes [10].

The aims of this study were to assess programme adherence, PA compliance, weight loss and other health risk factor changes in previously insufficiently active adults following two types of work-place interventions. The hypothesis was that both intervention arms would result in similar increased PA patterns and improved health profiles.

## 2. Materials and Methods

The workplace interventions were designed to incorporate daily PA in addition to nutrition education in order to encourage more active behaviour, healthy eating practices (results not reported here) and facilitate weight loss and were based primarily on a socio-ecological approach to behaviour change [11]. The after-work group followed a 40-day PA programme that was designed for insufficiently active adults and has been described previously [12],[13]. The pilot in-work programme was adapted from the 40-day PA programme. The 40-day PA group-based strategy required participants to attend instructor-led activities three times/week for 6 weeks (Monday, Wednesday, Friday) and undertake individual activities on all other days of the week. Participants maintained PA diaries recording their activity patterns including activity time, and average heart rate for all sessions undertaken. Health and fitness testing was conducted before and immediately after the 6-week programme.

University management fully supported this intervention trial and allowed staff to take time away from their office to participate. Following institutional ethical approval, email and poster advertisements were utilised to recruitment participants from a large university. Respondents received further information and completed the Active Australia PA questionnaire [14].

Selection criteria:

Participants had to:

(1) be “insufficiently active” according to the Active Australia criteria. (<150 min/wk of weighted PA; where vigorous activity minutes are doubled [14]) and or with a BMI ≥ 30 kg/m^2^.

(2) be willing to participate in either of the 40-day physical activity programs, or act as controls and be placed on a wait list.

(3) undergo a pre-exercise screening evaluation according to the Sports Medicine Australia (SMA) pre-exercise screening system [15].

(4) complete basic fitness measures [16].

Participants were excluded from the study if: (1) they were sufficiently active according to the Active Australia Questionnaire (i.e. ≥150 minutes of PA/week) or (2) based on the SMA screening guidelines, they were sent for medical clearance and clearance to participate was not provided.

**Figure 1.**
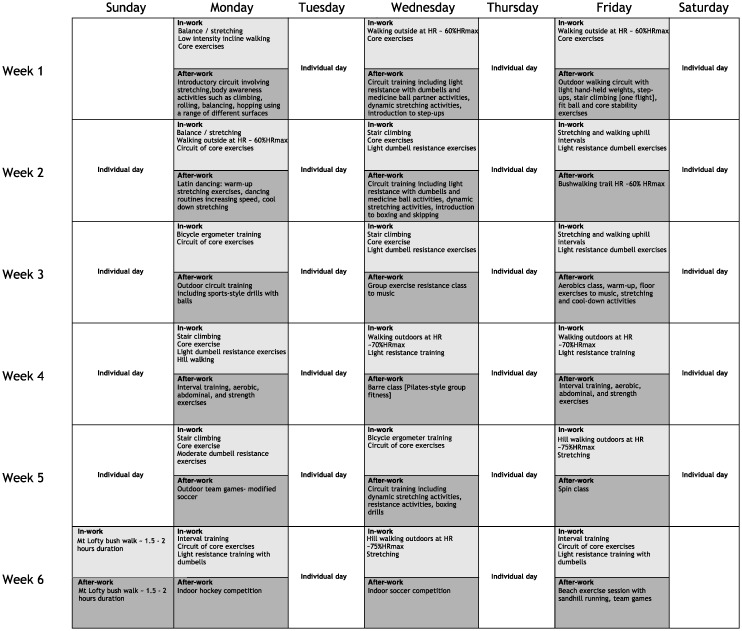
40-day physical activity programme. All instructor led exercise sessions were conducted on Monday, Wednesday and Friday. In-work participants undertook 2 × 15 minute sessions during the work day, after-work participants undertook a 60-minute session at the end of the work day.

Interventions: Participants were randomly allocated using a computer randomised sequence generator on a 1:1 ratio into one of three arms: (1) an after-work group that had 3 × 60 min instructor-led PA sessions each week of the six-week programme that mirrored our previously published group intervention (n = 25) [12]. Sessions were based primarily in the organisation's gymnasium and nearby outdoor playing fields and included a variety of resistance and aerobic-based activities; (2) an in-work group that had 2 × 15 min instructor-led PA periods 3 days/wk at mid-morning and mid-afternoon. Instructors collected participants from their work location and conducted circuit activities incorporating walking/stair-climbing, resistance training and callisthenic exercises (n = 23) for small groups (n ≤ 6 participants) in near-proximity to their office area. When participant numbers <3 for a group session that session was combined with another in close proximity; or (3) a wait-listed control group (n = 23) who were asked to maintain their usual PA. These participants undertook an after-work intervention following the post-testing. All intervention participants were required to accumulate and record a total of 60min/day PA across the 40-day programme. Participants were encouraged to start their individual PA program with walking and to include elements of the instructor-led sessions as they became more comfortable with the activities. All participants were provided with motivational tips on starting or increasing their daily PA for example: planning and preparing for PA the night before, being active with a friend, family member or pet, and creating motivational music lists to use during activities. Instructor-led sessions started conservatively and progressed in intensity across the duration of the programme (Figure 1).

The intervention participants were allocated heart rate (HR) monitors and diaries. HR monitors provided immediate feedback on exercise intensity during the sessions and were downloaded weekly for compliance checks with target PA time. Diaries were used to record daily PA types and issues associated with achieving target PA levels. Participants attended three nutrition workshops conducted by a nutritionist. These were based on the Australian Dietary Guidelines [17] and included energy balance, food groups, portion sizes, label reading, and healthy diet maintenance. Participants completed food diaries on two occasions, pre-intervention and week six of the intervention.

Physical activity patterns and health and fitness variables were evaluated both pre- and post-intervention. PA patterns over the previous week were assessed using the Active Australia Survey (AAS), a 7-day recall questionnaire that measures leisure-time PA participation [14]. Resting blood pressure (Dinamap Pro 100), anthropometric measures of height, weight, waist and hip girths [18], and fasting total cholesterol and glucose using finger-tip blood samples and a Reflotron Plus analyzer (Hoffman La Roche Ltd, Basel, Switzerland) were also collected [19]. A submaximal cycle ergometer test was undertaken that involved 3 × 3 minute stages to approximately 75% predicted HRmax [16]. This was used to estimate maximal aerobic fitness (VO_2max_). The Southern Adelaide Clinical Human Research Ethics Committee for Flinders University approved the study protocol and all participants gave informed written consent (HREC code 328.12).

Adherence required attendance at both pre- and post-testing sessions. Compliance was measured using intention to treat in two objective ways: (1) achieving a minimum of 60 min/day of recorded PA; and (2) attendance at the instructor-led sessions. Compliance was also determined using per protocol procedures whereby participants achieved ≥150 minPA/wk immediately post-intervention using the self-reported AAS [14].

Statistical analysis was performed using Statview software (Abacus Concepts Inc, CA). The study was powered to detect small to moderate effect size differences in fitness and PA patterns based on an alpha level of 5% and 80% power, taking into account an anticipated 10–15% dropout. Population proportion analysis was used to compare adherence among groups. Chi-square was used to assess differences in daily PA compliance. Comparisons in PA and health and fitness changes across study arms were made using repeat-measures ANOVA.

## 3. Results

**Figure 2.**
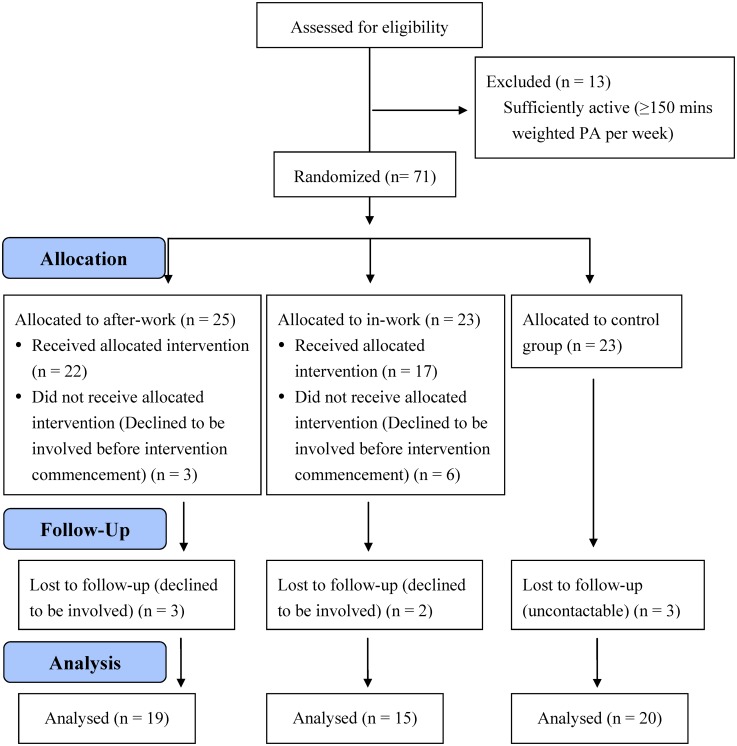
Flow diagram of the progress through the phases of the intervention.

Participant characteristics are shown in Table 1. All were recruited from the university community and consisted of administrative, clerical or academic staff. There were no differences in any variables between groups pre-intervention. Figure 2 outlines the flow of participants from the initial email enquiry through to final physiological testing at follow-up. Participants who declined to commence the intervention or did not return for follow-up were statistically no different to the participants who completed all components of the intervention.

Adherence was 87.0% for control participants compared with 76.0% and 65.2% for after-work and in-work participants, respectively. The adherence rates were not different among the three groups (*z* < 1.96). Compliance with the 60 min/day PA target was 52.3% and 18.9% for the after-work and in-work participants, respectively (*p* < 0.001). Attendance at group sessions was higher after-work (70.4%) versus in-work (26.4%), and higher compared to individual sessions (22.3% and 10.1%, respectively; *p* < 0.001). Using the AAS post-intervention showed ≥150 minPA/wk compliance levels were not different between the after-work and in-work completers (89.5% and 80.0%, respectively).

Table 2 shows pre-post intervention changes in PA, and health and fitness variables. Weighted PA levels increased in both groups. After-work participants increased more than in-work participants, with significantly more vigorous PA. Beneficial changes in BMI and aerobic fitness were significant in both intervention groups. Changes in BMI for the after-work participants were greater relative to controls while fitness improvements for the intervention participants exceeded those for the controls. Weight and glucose improvements were greater for the after-work versus in-work participants and controls. Waist and hip girth reductions were greater for after-work participants versus controls. No other changes were found relative to controls.

**Table 1.** Pre-intervention participant characteristics (mean ± SD).

	After-work n = 25	In-work n = 23	Control n = 23
Age (yr)	48.3 (± 11.2)	46.8 (± 7.9)	44.4 (± 9.3)
Females n (%)	22 (88%)	21 (91%)	19 (83%)
Total weighted PA (min/wk)	102 (± 81)	72 (± 92)	63 (± 48)
Vigorous PA (min/wk)	15 (± 23)	9 (± 18)	5 (± 13)
Height (cm)	166.3 (± 8.7)	165.6 (± 6.9)	169.8 (± 10.2)
Weight (kg)	81.8 (± 19.7)	81.9 (± 18.5)	78.2 (± 22.8)
BMI (kg/m^2^)	29.7 (± 7.7)	29.6 (± 5.2)	26.8 (± 6.1)
Waist (cm)	88.9 (± 16.8)	90.8 (± 12.9)	86.3 (± 17.2)
Total cholesterol (mmol/L)	4.74 (± 0.9)	5.0 (± 0.6)	4.88 (± 0.8)
Fasting BGL (mmol/L)	5.54 (± 1.0)	5.38 (± 1.6)	5.53 (± 0.4)
VO_2max_ (mL/kg/min)	25.1 (± 6.1)	24.1 (± 4.6)	27.8 (± 5.5)

**Table 2.** Health and fitness changes across the intervention for the three study arms.

Variable	After-work (n = 19)					In-work (n = 15)					Control (n = 20)					
	pre	SD	post	SD	Pre-post *p*	pre	SD	post	SD	Pre-post *p*	pre	SD	post	SD	Pre-post p	Intervention × time differences *p*
PA total weighted (min/wk)	110	81	705	366	<0.001	73	96	306	202	<0.001	69	48	140	131	0.021	<0.001 **^1,2,3^**
Vigorous PA (min/wk)	19	24	213	155	<0.001	13	20	71	85	0.013	5	13	17	34	0.106	<0.001 **^1,2^**
Weight (kg)	78.6	15.4	76.6	15.0	<0.001	84.7	20.6	84.1	20.3	0.128	79.8	23.5	80.2	23.5	0.246	<0.001 **^1,2,3^**
BMI (kg/m^2^)	28.0	5.8	27.2	5.5	<0.001	29.9	5.3	29.6	5.3	0.049	27.2	6.3	27.3	6.3	0.321	<0.001 **^2^**
Waist girth (cm)	85.9	13.9	82.6	11.5	0.019	91.6	14.5	89.9	13.8	0.064	88.2	17.1	88.3	17.4	0.815	0.031 **^2^**
Hip girth (cm)	107.0	10.1	105.7	9.1	0.013	109.6	10.6	108.6	11.0	0.062	103.7	12	104.0	12.7	0.513	0.046 **^2^**
Systolic BP (mmHg)	121	13	122	15	0.620	120	12	120	9	0.829	123	17	117	15	0.040	0.095
Diastolic BP (mmHg)	78	8	76	9	0.307	80	9	78	6	0.366	76	10	75	9	0.239	0.970
Total cholesterol (mmol/L)	4.85	1.0	4.47	0.9	0.005	5.10	0.6	4.92	0.5	0.096	4.93	0.9	4.71	0.8	0.012	0.364
Fasting BGL (mmol/L)	5.44	0.8	5.00	0.8	<0.001	5.00	0.3	4.87	0.3	0.126	5.57	0.4	5.52	0.5	0.517	0.006 **^1,2^**
VO_2max_ (mL/kg/min)	25.4	5.7	30.3	5.5	<0.001	23.2	3.7	26.9	3.8	<0.001	27.9	5.8	28.1	5.9	0.897	0.024 **^2,3^**

**Notes:** three study arms: **^1^** Significant difference between after-work and in-work, **^2^** Significant difference between after-work and controls, **^3^** Significant difference between in-work and controls. PA totals are weighted using vigorous minutes × 2.

## 4. Discussion

This study introduced insufficiently active and/or obese adults to PA and nutrition education interventions in the workplace. Comparisons were made between after-work and in-work programmes using outcome measures of adherence, compliance, PA behaviours, weight loss and other health risk factor changes.

Adherence rates following the six-week intervention were high and similar to our previous intervention [12],[13]. This is typical for short-term interventions and higher dropout rates are common as the programme duration increases [20].

In order to promote weight loss, 60 minutes of daily PA (420 min/wk) is recommended [5], although Australian PA guidelines suggest 150–300min/wk of moderate-intensity PA for general health benefits [4]. Compliance rates in the present study using the 60min/day threshold were almost 3-fold higher for the after-work participants compared to in-work participants. The attendance at the instructor-led sessions was also significantly higher for the after-work participants when compared to the in-work participants. The higher attendance rates for the after-work participants contributed positively to the additional PA patterns. Previous work has suggested that maintaining this level of PA is problematic [9] but an important element of the present study was to directly compare the efficacy of the intervention arms.

PA interventions using a socio-ecological model incorporate personal, social and environmental constructs that can range along a continuum from personal level factors to macro-environmental influences such as the physical and work environments [11]. There is strong to definitive evidence for the effectiveness of multi-component interventions in the workplace to reduce risk factors for chronic diseases [20]. Of relevance are programmes combining nutrition education and PA, and providing increased opportunity and places for PA such as organised sessions during work time [21],[22]. Therefore, such low in-work group attendance was unexpected. Additionally, compliance for the individual daily PA targets for weight loss (≥60 min/day) was also low for this intervention arm and lower than the after-work group. The total PA change, lower than expected weight loss and lower session attendance for the in-work group suggest the design was inferior to the after-work setting. During post-intervention testing, participants informally reported difficulties attending in-work sessions despite instructors meeting them in or near their office space. Participants described a range of issues such as clashes with scheduled meetings (despite the regular activity timetable), pressing work matters, and perceived problems with hygiene post PA. Despite these issues, compliance with the ≥150 min/wk target post-intervention was not different between the intervention arms (89.5 and 80.0%) and is reflected in other important health benefits for those completing the programmes. It appears that in-work participants compensated for the lower attendance rates by additional individual PA at other times although total levels were still significantly lower than the after-work group.

The rapid and dramatic increases in PA behaviours of between 233 and 595 weighted minutes of PA/wk resulted in health improvements in both intervention groups. However, the additional vigorous activity by the after-work participants is likely to account for the greater weight and anthropometric improvements, and benefits in cholesterol and BGL relative to the in-work group, as others have demonstrated [13],[23]–[25]. The aerobic fitness changes for both groups were impressive in such a short period but are typical given the low starting levels and large PA changes [26].

While it is not possible to determine the precise reasons for the differences in PA behaviour change between groups there are a number of important strategies that may help explain these results. Generally, previous work has shown a range of methodological characteristics, research design and psychological constructs may all impact behaviour change [11],[27]. This study combined the following elements to effect behaviour change: providing diaries, education sessions, technological support, pre-post testing, professional guidance and counselling, and improving self-efficacy can increase PA patterns [12],[13],[27]. Both intervention groups had a similar programme with the exception of the environment and timing of the exercise sessions. The brief nature of the in-work opportunities falls in line with guidelines that encourage people to take every opportunity to be active, even in shorter bouts of 10 minutes [28]. This fails to consider potential barriers such as those identified by our in-work participants including hygiene and disruption to work flow. The after-work approach in the current study overcame these barriers and it may be the same for dedicated sessions before work. This is not to be confused with incidental activity opportunities whereby even shorter breaks between sedentary behaviours, such as using the stairs between floors, walking between offices more often and regular standing breaks, have been shown to be important for health [29].

## 5. Limitations

The major limitations to this study were the relatively small participant numbers in this pilot and the short-term nature of the intervention. It is not known how long these short-term behaviour changes were maintained by the participants and this is an area for further research. Additionally, the potential impact that these PA interventions have on other health related behaviours was not measured. The majority of participants were female (87%) and this is typically seen in PA intervention studies [27]. A sampling protocol would have reduced the gender imbalance however this would have extended the time to recruit beyond the resources available to conduct the study. The results of this study apply to a well-educated, predominantly female, homogenous cohort in terms of socio-demographic indices. Therefore, the generalisability of this type of intervention is unknown in other workplaces.

## 6. Conclusions

In summary, both intervention strategies resulted in positive behaviour changes and associated health benefits. However, for in-work participants, the low attendance at the instructor-led sessions and overall low compliance with the weight loss targets show this was less successful than the after-work design. The results suggest this in-work intervention design is not optimal for weight loss within work places such as a tertiary institution.
